# Prevalence and determinants of anemia among children aged from 6 to 59 months in Liberia: a multilevel analysis of the 2019/20 Liberia demographic and health survey data

**DOI:** 10.3389/fped.2023.1152083

**Published:** 2023-04-18

**Authors:** Dagnew Getnet Adugna, Anteneh Ayelign Kibret, Hailu Aragie, Engidaw Fentahun Enyew, Gashaw Dessie, Mihret Melese, Wudneh Simegn, Endeshaw Chekol Abebe, Fitalew Tadele Admasu, Tadesse Asmamaw Dejenie

**Affiliations:** ^1^ Department of Human Anatomy, School of Medicine, College of Medicine and Health Science, University of Gondar, Gondar, Ethiopia; ^2^ Department of Biochemistry, School of Medicine, College of Medicine and Health Science, University of Gondar, Gondar, Ethiopia; ^3^ Department of Medical Physiology, School of Medicine, College of Medicine and Health Science, University of Gondar, Gondar, Ethiopia; ^4^ Department of Social and Administrative Pharmacy, School of Pharmacy, University of Gondar, Gondar, Ethiopia; ^5^ Department of Biochemistry, College of Medicine and Health Science, Debre Tabor University, Debre Tabor, Ethiopia

**Keywords:** anemia, prevalence, determinants, children, Liberia

## Abstract

**Background:**

Anemia is a serious worldwide public health issue that happens at any stage of life but primarily affects young kids and pregnant mothers. Although anemia has a significant impact on child health, its magnitude and associated factors in children aged 6–59 months have not been yet studied in Liberia. Therefore, the aim of this study was to identify the prevalence and determinants of anemia in children aged 6–59 months in Liberia.

**Methods:**

The data was extracted from Liberia Demographic and Health Survey, conducted from October 2019 to February 2020. The sample was obtained using a stratified two-stage cluster sampling technique. An overall weighted sample of 2,524 kids aged 6–59 months was involved in the final analysis. We used Stata version 14 software for data extraction and analysis. A multilevel logistic regression model was employed to identify factors associated with anemia. Variables with a *P*-value of <0.2 in the bivariable logistic regression analysis were selected as candidates for multivariable analysis. In multivariable analysis, the adjusted odds ratios (AOR) with the 95% confidence interval (CI) were declared as the determinants of anemia.

**Results:**

The prevalence of anemia in children aged 6–59 months in Liberia was 70.8% [95% CI: 68.9%, 72.5%]. Of these, 3.4% were severe anemia, 38.3% were moderate anemia and 29.1% were mild anemia. Children aged 6–23 and 24–42 months, being stunted, children from households with unimproved toilet facilities, children from households with unimproved water sources, and lack of media (television) exposure were significantly associated with higher odds of anemia. However, using mosquito bed nets, living in the Northwestern and Northcentral region were significantly associated with lower odds of anemia among children 6–59 months.

**Conclusion:**

In this study, anemia in kids aged 6–59 months in Liberia was a main public health issue. Age of the child, stunting, toilet facility, water source, exposure to television, mosquito bed net use, and region were significant determinants of anemia. Therefore, it is better to provide intervention for the early detection and management of stunted children. Similarly, interventions should be strengthened to address unimproved water sources, unimproved toilet facilities, and lack of media exposure.

## Background

Anemia is a disorder that is marked by an inadequate number of healthy erythrocytes, usually accompanied by low levels of hemoglobin or defective blood cell architecture, which hinders the blood from adequately providing oxygen to the body's organs (1). Based on the World Health Organization (WHO), hemoglobin levels below 11.0 g/dl are considered anemia in under-five years old children (2). Besides, anemia considering a serious public health concern when its prevalence is 40% or above (3, 4). Children are more susceptible to anemia during the childhood period because it is the crucial period for growth and development (5).

Anemia is a serious worldwide public health issue that happens at any stage of life but primarily affects young kids and pregnant mothers (6–9). Worldwide, over 293.2 million (47.3%) children suffer from anemia and most (67.6%) of them reside in Africa (7, 10–12). The highest prevalence (64.1%) of anemia in kids aged 5–59 months is found in Sub-Saharan Africa (13). Anemia in children can have negative short- and long-term health effects. It has a significant impact on children, affecting things like low educational performance, low immunity, decreased physical and behavioral growth, and vulnerability to infection (14–17).

Although the etiologies of anemia are multifactorial, nutritional deficiencies (like iron, folic acid, vitamin B12, and A) and communicable diseases [like tuberculosis, malaria, hookworm, and human immune virus (HIV) infections] are the most common causes of childhood anemia (18–20). Previous studies indicated that several factors are related to anemia in under-5 kids. These include the age of mothers (21), child age (9, 22, 23), child sex (13), malnutrition (3, 13, 24), household wealth status (13, 21), lack of improved water source (25–27), unimproved toilet facility (25–27), lack of media exposure (28–30), residence (31), maternal anemia (32, 33), maternal education (34), infections (malaria and hookworm) (35), birth order (13), large family members (3, 13), and region (3). Additionally, in low-income countries, anemia may vary from country to country by socioeconomic factors (7, 33).

Although several interventions made to date through the governments of low-income countries and other relevant stakeholders, childhood anemia remains a serious public health issue (36–39). Although anemia has a significant impact on the health of children, its magnitude and associated factors in children aged 6–59 months have not been yet studied in Liberia. Therefore, the aim of this study was to identify the prevalence and determinants of anemia in children aged 6–59 months in Liberia. We hypothesized that the burden of anemia in kids aged 6–59 months in Liberia is high and several risk factors are correlated with anemia. The results of the current study will aid program managers in generating good decisions and designing a proper intervention to compact this serious public health problem in Liberia.

## Methods

### Data source and sampling procedure

The data was extracted from the fifth Liberia Demographic and Health Survey (LDHS-V) conducted from October 16, 2019, to February 12, 2020. A stratified two-stage cluster sampling technique was used to select the study participants using the 2008 national population and housing census (NPHC) as a sampling frame. Liberia is initially split into fifteen counties, which are then organized into five geographical areas, each of which consists of three counties. Every county was split into districts. Then, every district was further divided into clans. Every clan was divided into enumeration zones in the 2008 NPHC. Based on the Liberian census frame, each enumeration area has an average of 100 households. An overall of 325 clusters were selected in the 1st stage using a stratified two-stage cluster sampling technique. In the 2nd stage, a fixed number of thirty households for each cluster were picked using a systematic selection method with an equal chance of success. Then, hemoglobin level testing was done in kids aged between 6 and 59 months in the chosen households. Blood samples were taken from a finger prick or a heel prick and collected in a microcuvette and which was then analyzed by a battery-operated portable HemoCue 201 + analyzer that shows hemoglobin level. Finally, anemia was defined according to the hemoglobin concentration. LDHS consists of different datasets such as men, women, children, birth, and household datasets. In this study, we extract the dependent and independent variables using the PR file. From the overall of 3,225 under-five children, a total weighted sample of 2,524 kids aged between 6 and 59 months was involved in the study. Data on hemoglobin levels from the survey were available for 2,907 kids.

### Measurement of variables

#### Outcome variables

The response variable for this study was the anemic status of kids aged between 6 and 59 months which were coded as 1 for “yes” and 0 for “no”. Based on WHO guidelines, kids aged between 6 and 59 months are considered anemic if their hemoglobin concentrations are less than 11.0 g/dl. Moreover, anemia was categorized as severe anemia (if hemoglobin level below 7.0 g/dl), moderate (hemoglobin level 7.0–9.9 g/dl), and mild (hemoglobin concentration 10.0 g/dl to 10.9 g/dl).

#### Independent variables

In the present study, the individual-level and community-level factors were grouped as explanatory variables. The explanatory variables were further categorized as household, parental, child, and community-related characteristics. Household-related factors were family size (categorized as ≤4, 5–8, and ≥9), household head sex (male and female), number of under-five kids (≤1 and more than 1), use of bed net, media exposure (yes and no), toilet facilities (improved and non-improved), water source (improved and non-improved), and household wealth status (poor, middle, and rich). According to LDHS 2019–20; bottled water, public taps, rainwater, tube wells, hand pumps, piped water, standpipes, boreholes, protected dug wells and springs, and water delivered *via* a tanker truck or a cart with a small tank are considered to be improved sources of drinking water. Additionally, pit latrines with slabs, composting toilets, pit latrines, or unknown destination, flush, or pour-flush toilets that flush water and waste to a piped sewer system, septic tank, and ventilated improved pit latrines are considered to be improved toilet facilities. Child-related factors were the age of a child (6–23 months, 24–42 months, and 43–59 months), child sex (male and female), birth order (<5 and ≥5), wasting status, underweight status, and stunting status. Stunting is described as the kids with height for age *Z*-score less than minus two standard deviations (< −2SD). Wasting is described as the kids with weight for height *Z*-score below −2SD and underweight are described as the kids with weight for age *Z*-score less than −2SD. Women's educational status (no formal education, elementary education, and secondary and above), mother alive (yes and no), and father alive (yes and no) were assessed as parental-related factors. Lastly, the community-related variables were residence (urban and rural) and region (Northwestern, Southcentral, Southeastern-a, Southeastern-b, and Northcentral).

### Statistical analysis

We used Stata version 14 software to extract and analyze the data. Descriptive statistics were calculated and the finding was presented in tables, figures, and text. Prior to conducting any statistical analysis, the data were weighted to improve the national representativeness of the survey results and to obtain accurate estimates and standard errors. A multilevel binary logistic regression model was performed to determine associated factors of anemia to consider the hierarchical nature of LDHS data. In LDHS, kids and women nested within a cluster and we hypothesize that participants in a similar cluster are more likely to share similar characteristics than participants in another cluster. This disrupts the independent assumptions of the standard logistic regression model like the independent observations and equal variance assumptions. This indicates the need to take into account the heterogeneity between clusters by using an advanced model. Thus, 4 models have developed; the 1st model or the null model contains only the dependent variable, the 2nd model, or Model I contained only the individual-level factors, the 3rd model, or Model II had only community-level factors, and the last model, or model III contained both individual and community-level factors. To determine whether there was clustering or not, the Intraclass Correlation Coefficient (ICC) and Median Odds Ratio (MOR) were done. Deviance [−2 log-likelihood (LL)] and the Proportional Change in Variance (PCV) were used to compare the models because both models were nested, and the lowest deviance model was selected for reporting and interpreting findings. Both bivariable and multivariable logistic regression model was employed to determine the associated factors of anemia in children aged 6–59 months. Variables with a *P*-value of less than 0.2 in the bivariable analysis were chosen as candidates for multivariable regression analysis. Variables with a *P*-value of <0.05 were considered significant factors associated with childhood anemia in multivariable analysis. Finally, an adjusted odds ratio (AOR) with a 95% confidence interval (CI) was calculated to assess the strength and direction of the associations.

## Results

### Descriptive characteristics of children aged 6–59 months in Liberia

An overall of 2,524 children aged 6–59 months were included in this study. Of these, 41.8% were from Southcentral, 36.7% from Northcentral, 9% from Northwestern, and 6.8% from the southeastern-a region. Besides, around 51.9% were from rural areas. About 34.1% of children were aged 24–42 months and 51% were females. Concerning nutritional status, 3.3%, 30.5%, and 11.4% of the children were wasted, stunted, and underweight, respectively. Regarding family size and educational status, about (54.1%) of respondents were from families' with 5–8 members, and 36.5% of mothers had primary education. Majorities (82.2%) of participants were from households with an improved water source and 53.2% of respondents were from households with non-improved toilet facilities (Table 1).

**Table 1:** Descriptive characteristics of study participants in Liberia (*N* = 2,524).

Variables	Categories	Frequency (*N* = 2,524)	Percentage (%)
**Child's characteristics**			
Age of child (in months)	6–23	843	33.4
	24–42	860	34.1
	43–59	821	32.5
Sex of child	Male	1,238	49
	Female	1,286	51
Birth order	<5	1,455	58
	≥5	1,069	42
Wasting	Yes	83	3.3
	No	2,441	96.7
Stunting	Yes	770	30.5
	No	1,754	69.5
Underweight	Yes	287	11.4
	No	2,337	88.6
**Parental-related characteristics**			
Mothers educational status	No formal education	775	30.7
	Primary education	921	36.5
	Secondary and above	828	32.8
Mother alive	No	20	0.8
	Yes	2,504	99.2
Father alive	No	68	2.7
	Yes	2,456	97.3
**Household characteristics**			
Household wealth status	Poor	1,187	47.1
	Middle	506	20.0
	Rich	831	32.9
Number of household members	≤4	633	25.1
	5–8	1,365	54.1
	≥9	526	20.8
Source of drinking water supply	Improved	2,075	82.2
	Non-improved	449	17.8
Toilet facilities	Improved	1,180	46.8
	Non-improved	1,344	53.2
Sex of household head	Male	1,603	63.5
	Female	921	36.5
Number of under-5 children's	≤1	1,077	42.7
	>2	1,447	57.3
Media (Television) exposure	No	2,083	82.5
	Yes	441	17.4
Radio	No	1,393	55.2
	Yes	1,131	44.8
Mosquito bed net for sleeping	No	968	38.4
	Yes	1,556	61.6
**Community-level characteristics**			
Residence	Urban	1,311	51.9
	Rural	1,213	48.1
Region	Northwestern	226	9.0
	Southcentral	1,055	41.8
	Southeastern a	172	6.8
	Southeastern b	144	5.7
	Northcentral	927	36.7

### Prevalence of anemia among children aged 6–59 months in Liberia

The total prevalence of anemia among kids aged 6–59 months in Liberia was 70.8% [95% CI: 68.9%, 72.5%] with the highest burden in the Northwestern (77.2%) and lowest in Northcentral (68.8%) region. The study also indicated that 3.4% [95% CI: 2.8%, 4.2%] of kids aged 6–59 months had severe anemia, 38.3% [95% CI: 36.3%, 40.1%] moderate anemia, and 29.1% [95% CI: 27.4%, 30.9%] mild anemia. The highest burden of anemia was found in kids who were stunted. Concerning the severity of anemia, the highest burden of severe anemia was found in children who were wasting (5.9%). The prevalence of anemia was higher in younger children aged 6–23 months (75.5%) compared with older children aged 36–59 months (61.5%). Among young children aged 6–23 months, 3.3%, 43%, and 29.4% of them were severe, moderate, and mild anemia, respectively (Table 2).

**Table 2:** The prevalence and severity of anemia based on the child-related, household, paternal, and community-level characteristics in Liberia (*N* = 2,524).

Variable	Categories	Anemia status and severity level (%)				Overall anemia prevalence (%)
		Severely anemic	Moderately anemic	Mild anemic	No anemic	
Age of child (in months)	6–23	3.3	43	29.4	24.3	75.7
	24–42	4.4	40.4	30.0	25.2	74.8
	43–59	2.6	31.1	27.8	38.5	61.5
Sex of child	Male	3	38.4	29.3	29.2	70.8
	Female	3.8	38.1	28.9	29.2	70.8
Under-weight	Yes	5.7	48.0	20.8	25.5	74.5
	No	3.1	37.0	30.2	29.7	70.3
Stunting	Yes	4.5	46.1	27.0	22.4	77.6
	No	3	34.8	30	32.2	67.8
Wasting	Yes	5.9	38.3	22.0	33.8	66.2
	No	3.3	38.2	29.4	29.1	70.9
Women education	No formal education	3.7	36.7	26.6	33.0	67
	Primary education	3.7	41.9	28.6	25.8	74.2
	Secondary and above	2.9	35.5	32.1	29.5	70.5
Water source	Improved	3.2	38.8	29.4	28.6	71.4
	Non-improved	4.3	35.6	28.0	32.1	67.9
Toilet facilities	Improved	3.6	31.8	31.9	32.7	67.3
	Non-improved	3.3	43.8	26.7	26.2	73.8
Wealth index	Poor	4.4	39.3	27.3	29.0	71.0
	Middle	3.2	41.6	31.7	23.5	76.5
	Rich	2.2	34.6	30.1	33.1	66.9
Residence	Urban	3.0	37.0	30.1	29.9	70.1
	Rural	3.9	39.6	28.1	28.4	71.6
Region	Northwestern	3.6	43.7	24.9	27.8	77.2
	Southcentral	3.6	38.9	28.7	28.8	71.2
	Southeastern a	3.0	38.4	30.3	28.3	71.7
	Southeastern b	4.1	47.6	24.8	23.5	76.5
	Northcentral	3.1	34.6	31.1	31.2	68.8
Overall prevalence (95% CI)		3.4 [2.8, 4.2]	38.3 [36.3, 40.1]	29.1 [27.4, 30.9]	29.2 [27.5, 30]	70.8 [68.9, 72.5]

### Random effect analysis result and model fitness

In this study, the random-effect model analysis was checked using ICC, MOR, and PCV. In the null model, the ICC value was 0.103; showing that 10.3% of the overall variation of anemia in kids aged 6–59 months was because of variations between clusters or communities but the rest undetermined 89.7% of the overall variability of anemia was due to the individual variations. In addition, the highest MOR value was 1.79 in the 1st model, indicating that there was significant clustering of anemia in children aged 6–59 months. Furthermore, the highest PCV value (0.13) in the last model indicated that nearly 13% of the variation in anemia was explained by both individual and community-level variables. Concerning model fitness, model III, which includes both individual and community-level variables, was the best-fitted model for the data because it has the lowest deviance value (3,051.32) (Table 3).

**Table 3:** Random effect model and model fitness for the assessment of anemia among children aged 6–59 months in Liberia.

Parameter	Null model	Model I	Model II	Model III
Community level variance	0.38 (0.23, 0.61)	0.37 (0.23, 0.61)	0.35 (0.20, 0.57)	0.33 (0.19, 0.56)
ICC	0.103	0.092	0.095	0.091
MOR	1.79	1.78	1.74	1.72
PCV	Ref	0.014	0.085	0.130
Model comparison				
LLR	−1,582.85	−1,531.29	−1,578.93	−1,525.66
Deviance (−2LLR)	3,165.70	3,062.58	3,157.86	3,051.32

ICC, intra-class correlation coefficient; LLR, log-likelihood ratio; MOR, median odds ratio.

### Determinants of anemia in children aged 6–59 months in Liberia

We used model III (best-fitted model) to identify the associated factors of anemia in kids aged 6–59 months in Liberia. Bivariable analysis was done to identify the significant factors of anemia. Accordingly, individual-level factors [such as the age of the child, stunting, underweight, household wealth status, toilet facility, source of water, exposure to media (television), and mosquito bed net use] and community-level factors (region) had a *P*-value of less than 0.2 in the bivariable analysis and were considered for the multivariable regression analysis. However, in the multivariable regression model; individual-level variables like the age of the child, stunting, toilet facility, source of drinking water, exposure status to media (television), and mosquito bed net use were significant factors associated with anemia. Among community-level factors, the region was a significant determinant of anemia in kids.

The odds of having anemia in kids aged 6–23 and 24–42 months were 2.4 times [adjusted odds ratio (AOR) = 2.4; 95%CI = 1.9, 3.0] and 1.8 times [AOR = 1.8; 95% CI 1.5, 2.3] higher than those of kids aged 43–59 months, respectively. The odds of developing anemia in stunted children were 1.6 times [AOR = 1.6; 95%CI = 1.3, 1.9] higher than their counterparts. Kids from households with unimproved water sources were 1.7 times [AOR = 1.7; 95%CI = 1.6, 1.8] higher odds of anemia than those kids from households with improved water sources. Similarly, children from households with unimproved toilet facilities were 1.3 times [AOR = 1.3; 1.1, 1.6] higher chances of anemia than those children from households with improved toilet facilities. Our study showed that children from households who had no media (television) exposure were 1.6 times [AOR = 1.6; 95% CI = 1.1, 2.4] a higher chance of developing anemia compared to their counterparts. Children from households who used mosquito bed nets for sleeping had 20% [AOR = 0.8; 95% CI = 0.6, 0.9] lower chances of having anemia than those who do not use bed nets. The odds of anemia were 0.6 [AOR = 0.6; 95% CI = 0.4, 0.8] and 0.7 [AOR = 0.7; 95%CI = 0.5, 0.9] times lower in children who were living in Northwestern and Northcentral, respectively, than children from South eastern-b region (Table 4).

**Table 4:** Individual and community-level factors associated with anemia among children aged 6–59 months in Liberia, 2019/20 (*N* = 2,524).

Variable	Categories	Anemia		COR (95% CI)	AOR (95% CI)
		Yes	No		
Age of child	6–23	638	205	2.3 (1.8, 2.9)**	2.4 (1.9, 3.0)**
	24–42	643	217	1.9 (1.5, 2.3)**	1.8 (1.5, 2.3)**
	43–59	505	316	1	1
Stunting	Yes	598	173	1.5 (1.2, 1.8)**	1.6 (1.3, 1.9)**
	No	1,188	565	1	1
Underweight	Yes	213	73	1.3 (0.98, 1.7)	0.9 (0.9, 1.3)
	No	1,573	665	1	1
Household wealth status	Poor	843	344	1.0 (0.7, 1.3)	0.8 (0.6, 1.2)
	Middle	387	119	1.4 (1.1, 1.8)	1.1 (0.8,1.6)
	Rich	556	275	1	1
Source of drinking water	Improved	1,481	594	1	1
	Non-improved	305	144	1.8 (1.6, 1.9)*	1.7 (1.6, 1.8)*
Toilet facilities	Improved	794	386	1	1
	Non-improved	992	352	1.4 (1.2, 1.6)*	1.3 (1.1, 1.6)*
Mosquito bed net use	Yes	1,092	464	0.8 (0.6,0.9)*	0.8 (0.6, 0.9)*
	No	694	274	1	1
Media exposure (television)	Yes	285	156	1	1
	No	1,501	582	1.5 (1.1, 2.0)*	1.6 (1.1,2.4)**
Region	Northwestern	163	63	0.6 (0.4,0.9)*	0.6 (0.4, 0.8)*
	Southcentral	752	303	0.9 (0.6,1.3)	0.9 (0.6, 1.4)
	South eastern-a	123.	49	0.8 (0.5,1.2)	0.8 (0.5, 1.2)
	South eastern-b	110	34	1	1
	Northcentral	638	289	0.7 (0.5, 0.9)*	0.7 (0.5, 0.9)*

COR, crude odds ratio; AOR, adjusted odds ratio; CI, confidence interval, 1, reference.

*P-value < 0.05.

**P-value < 0.01.

## Discussion

This study aimed to determine anemia and its determinants in children aged 6–59 months. The total prevalence of anemia among children aged 6–59 months in Liberia was 70.8% [95% CI: 68.9%, 72.5%], implying that anemia among children remains a main public health issue in Liberia. Despite the joint approaches mainly iron supplementation and communicable disease control including helminth and malaria treatments are being provided *via* the WHO to reduce the burden of anemia, it is still a serious public health concern for children in Liberia. The prevalence of anemia in our study is similar to the study conducted in Togo (40). However, it is greater than the previous studies done in Sub-Saharan Africa (13, 41), Brazil (42), Europe (43), and Ecuador (44). The first reason for this may be because of the high burden of chronic malnutrition in under-five children following inadequate dietary intake of nutrients, in developing countries (45, 46). Secondly, because of their repeated exposure to unsanitary conditions and environments that promote the transmission and spread of parasites, children in low-income countries particularly in Liberia are greatly vulnerable to communicable diseases like malaria, hookworms, schistosomasis, and visceral leishmaniasis (47–49). The third explanation could be due to children in Liberia do not have access to basic healthcare services due to political and economic instability brought on by devastating civil wars and the Ebola epidemic.

In the final model, we found that being younger age of a child, being stunted, being from households without improved toilet facilities, children from households without improved water sources, and lack of media (television) exposure were significantly associated with higher chances of anemia. However, using mosquito bed nets, living in the Northwestern and Northcentral region were significantly associated with lower odds of anemia in children 6–59 months of age.

In our study, age was significantly associated with childhood anemia. Children aged 6–23 months and 24–43 months had higher odds of anemia than children aged 43–59 months old. This result is in agreement with other previous studies conducted in Ethiopia (50, 51), Bangladesh (31), and Sub-Saharan Africa (13). This might be due to a high need for iron caused by children's early-life rapid growth and development, which increases iron consumption (52). Additionally, the age range of 6–23 months is crucial for beginning complementary feeding and has a high risk of food and water contamination, which may enhance the occurrence of diseases like typhoid, hookworm, ascariasis, amoebiasis, and giardiasis that are transmitted through contaminated water and food (53).

This study revealed that stunted children had higher chances of developing anemia than normal children. The present finding is in line with other studies conducted elsewhere (13, 51, 54). The primary probable reason might be both stunting and childhood anemia are caused by malnutrition, and therefore follow a common causal pathway which is; feeding children <4 times per day and having low dietary diversity. The second explanation is that dietary deficiency may decrease immunity and lead to recurrent infection, which in turn reduces iron storage. In addition to the deficit of micronutrients required for erythropoiesis, low nutritional status is associated with impaired immunity, and, thus, infections and intestinal infestations also have additive effects of micronutrient deficiencies for causing anemia (55). Furthermore, malnourished kids are more liable to a lack of micronutrients like folic acid, vitamin B12, and iron, which are essential to the synthesis of hemoglobin and DNA during the formation of erythrocytes, as a result, contribute to the onset of anemia (55).

The study showed that being from households with non-improved toilet facilities and water sources were significantly associated with higher odds of anemia. These are supported by previous reports done elsewhere (25–27, 56–58). It might be because children who use unimproved toilet facilities and water sources are more likely to develop waterborne and foodborne diseases, which could raise the incidence of anemia. Furthermore, young kids are vulnerable to intestinal infections for example hookworm, which is the leading cause of anemia in unsanitary environments (59).

Our study also found that children's from households who had no exposed media (television) had a higher chance of having anemia than their counterparts. The result is supported by other studies conducted in Ethiopia (60) and Lao People's Democratic Republic (30). This could as a result of the media serving as the primary source of information and raising knowledge about the causes of anemia as well as effective child-feeding practices to minimize the burden.

The finding of this study showed that kids who use mosquito bed nets at night had lower odds of developing anemia compared with those who did not use bed nets which is supported by the study reported in Ethiopia (61) and Malawi (62, 63). The rationale might be that kids who sleep under mosquito bed nets are better protected from *Anopheles mosquito* bites, which can reduce the incidence of malaria and, as a result, anemia.

Furthermore, the region was significantly associated with anemia in this study. The risk of developing anemia was lower in kids who were living in Northwestern and Northcentral, respectively than in kids from South eastern-b region**.** The result is similar to previous reports from Ghana and Ethiopia (51, 64). Even though variations between regions in Liberia were not fully investigated, data from similar low-income countries revealed a geographical variation in anemia (65, 66). This might be suggested by variations in anemia risk in the spread of infectious diseases related to the food supply, regional geographic backgrounds, accessibility and availability of a variety of diets, and use of health facilities (67). Besides, it could be because of the difference in socioeconomic background, living conditions, and cultural beliefs on feeding practices across regions.

## Strength and limitations

This study was conducted at a national level with a huge sample size and proper statistical analysis considering the hierarchical nature of the DHS data. Thus, we firmly believe that it gives more accurate and generalizable information for policymakers and program managers to develop intervention strategies for the problem at the national level.

However, the study has some limitations: the cross-sectional nature of DHS data does not permit cause-and-effect relationships to be established between explanatory variables and anemia. We didn't include some explanatory variables like dietary factors, parasitic infection (e.g., childhood malaria and hookworm) and chronic illness (e.g., maternal anemia) in the study since these factors are not found in the LDHS data. Therefore, a further prospective follow-up study should be conducted to address such factors.

## Conclusion

The prevalence of anemia in children aged 6–59 months in Liberia was relatively higher than in previous reports, indicating that it is a main public health issue. We found that the age of a child, stunting, toilet facility, source of drinking water, exposure status to media (television), mosquito bed net use, and region were significant determinants of anemia among children. Therefore, it is better to provide interventions such as community-based screening for early detection and management of kids with stunting to decrease the burden of anemia. It is also better to give special attention to children <24 months of age. Besides, providing special emphasis to those children who had higher odds of anemia like children who are from households with non-improved toilet facilities, non-improved water sources, and lack of media exposure is recommended.

## Data Availability

Publicly available datasets were analyzed in this study. This data can be found here: https://dhsprogram.com/Data/.

## References

[1] Gao J Monaghan SA. Red blood cell/hemoglobin disorders. In: Hematopathology. Elsevier (2018). p. 3–56.e2.

[2] World Health Organization. Iron deficiency anemia. Assessment, prevention, and control. In: A guide for programme managers. Geneva: World Health Organization (2001). p. 47–62.

[3] Asresie MB Fekadu GA Dagnew GW. Determinants of anemia among children aged 6–59 months in Ethiopia: further analysis of the 2016 Ethiopian demographic health survey. Adv Public Health. (2020) 2020:1–6.

[4] WHO U. UNU. Iron deficiency anaemia: Assessment, prevention and control, a guide for programme managers. Geneva: World Health Organization (2001). WHO/NHD/01.3, 2015.

[5] Ramakrishnan U. Functional consequences of nutritional anemia during pregnancy and early childhood. Boca Raton, Florida: CRC Press, LLC (2001).

[6] Milman N. Anemia—still a major health problem in many parts of the world! Ann Hematol. (2011) 90(4):369–77. 10.1007/s00277-010-1144-5 21221586

[7] Stevens GA Finucane MM De-Regil LM Paciorek CJ Flaxman SR Branca F Global, regional, and national trends in haemoglobin concentration and prevalence of total and severe anaemia in children and pregnant and non-pregnant women for 1995–2011: a systematic analysis of population-representative data. Lancet Glob Health. (2013) 1(1):e16–25. 10.1016/S2214-109X(13)70001-9 25103581 PMC4547326

[8] Unicef U, WHO U. WHO: iron deficiency anaemia: assessment, prevention, and control. In: A guide for programme managers. Geneva: World Health Organization (2001).

[9] Seifu BL Tesema GA. Individual-and community-level factors associated with anemia among children aged 6–23 months in Sub-Saharan Africa: evidence from 32 Sub-Saharan African countries. Arch Public Health. (2022) 80(1):1–12. 10.1186/s13690-022-00950-y 35933419 PMC9357302

[10] Pasricha S-R. Anemia: a comprehensive global estimate. Blood. (2014) 123(5):611–2. 10.1182/blood-2013-12-543405 24482500

[11] Gebreweld A Ali N Ali R Fisha T. Prevalence of anemia and its associated factors among children under five years of age attending at guguftu health center, South Wollo, Northeast Ethiopia. PLoS One. (2019) 14(7):e0218961. 10.1371/journal.pone.0218961 31276472 PMC6611584

[12] De Benoist B Cogswell M Egli I McLean E. Worldwide prevalence of anaemia 1993–2005; WHO global database of anaemia (2008). 10.1017/S1368980008002401 18498676

[13] Tesema GA Worku MG Tessema ZT Teshale AB Alem AZ Yeshaw Y Prevalence and determinants of severity levels of anemia among children aged 6–59 months in Sub-Saharan Africa: a multilevel ordinal logistic regression analysis. PLoS One. (2021) 16(4):e0249978. 10.1371/journal.pone.0249978 33891603 PMC8064743

[14] Brabin BJ Premji Z Verhoeff F. An analysis of anemia and child mortality. J Nutr. (2001) 131(2):636S–48S. 10.1093/jn/131.2.636S 11160595

[15] McCann JC Ames BN. An overview of evidence for a causal relation between iron deficiency during development and deficits in cognitive or behavioral function. Am J Clin Nutr. (2007) 85(4):931–45. 10.1093/ajcn/85.4.931 17413089

[16] Sachdev H Gera T Nestel P. Effect of iron supplementation on mental and motor development in children: systematic review of randomised controlled trials. Public Health Nutr. (2005) 8(2):117–32. 10.1079/PHN2004677 15877905

[17] Balarajan Y Ramakrishnan U Özaltin E Shankar AH Subramanian S. Anaemia in low-income and middle-income countries. Lancet. (2011) 378(9809):2123–35. 10.1016/S0140-6736(10)62304-5 21813172

[18] Chaparro CM Suchdev PS. Anemia epidemiology, pathophysiology, and etiology in low-and middle-income countries. Ann N Y Acad Sci. (2019) 1450(1):15–31. 10.1111/nyas.14092 31008520 PMC6697587

[19] Jonker FA van Hensbroek MB. Anaemia, iron deficiency and susceptibility to infections. J Infect. (2014) 69:S23–S7. 10.1016/j.jinf.2014.08.007 25264159

[20] Dreyfuss ML Stoltzfus RJ Shrestha JB Pradhan EK LeClerq SC Khatry SK Hookworms, malaria and vitamin A deficiency contribute to anemia and iron deficiency among pregnant women in the plains of Nepal. J Nutr. (2000) 130(10):2527–36. 10.1093/jn/130.10.2527 11015485

[21] Sanchez JM Vicario I Albizuri J Guraya T Acuña EM. Design, microstructure and mechanical properties of cast medium entropy aluminium alloys. Sci Rep. (2019) 9(1):1–12. 10.1038/s41598-018-37186-2 31043686 PMC6494811

[22] Li H Xiao J Liao M Huang G Zheng J Wang H Anemia prevalence, severity and associated factors among children aged 6–71 months in rural hunan province, China: a community-based cross-sectional study. BMC Public Health. (2020) 20(1):1–13. 10.1186/s12889-019-7969-5 32576157 PMC7310416

[23] Habte D Asrat K Magafu MG Ali IM Benti T Abtew W Maternal risk factors for childhood anaemia in Ethiopia. Afr J Reprod Health. (2013) 17(3):110–8. https://hdl.handle.net/10520/EJC140167 24069773

[24] Engidaye G Melku M Yalew A Getaneh Z Asrie F Enawgaw B. Under nutrition, maternal anemia and household food insecurity are risk factors of anemia among preschool aged children in menz gera midir district, Eastern Amhara, Ethiopia: a community based cross-sectional study. BMC Public Health. (2019) 19(1):1–11. 10.1186/s12889-019-7293-0 31324244 PMC6642588

[25] Yu EX Addo OY Williams AM Engle-Stone R Ou J Huang W Association between anemia and household water source or sanitation in preschool children: the biomarkers reflecting inflammation and nutritional determinants of Anemia (BRINDA) project. Am J Clin Nutr. (2020) 112(Suppl 1):488S–97S. 10.1093/ajcn/nqaa148 PMC7396266 32743647

[26] Teshale AB Tesema GA Worku MG Yeshaw Y Tessema ZT. Anemia and its associated factors among women of reproductive age in Eastern Africa: a multilevel mixed-effects generalized linear model. PLos One. (2020) 15(9):e0238957. 10.1371/journal.pone.0238957 32915880 PMC7485848

[27] Nankinga O Aguta D. Determinants of Anemia among women in Uganda: further analysis of the Uganda demographic and health surveys. BMC Public Health. (2019) 19(1):1–9. 10.1186/s12889-019-8114-1 31888579 PMC6937990

[28] Alemu T Umeta M. Reproductive and obstetric factors are key predictors of maternal anemia during pregnancy in Ethiopia: evidence from demographic and health survey (2011). Anemia. (2015) 2015:1–8. 10.1155/2015/649815 PMC4568321 26417454

[29] Mbule MA Byaruhanga YB Kabahenda M Lubowa A. Determinants of anaemia among pregnant women in rural Uganda. Rural Remote Health. (2013) 13(2):1–15. 23679828

[30] Keokenchanh S Kounnavong S Tokinobu A Midorikawa K Ikeda W Morita A Prevalence of anemia and its associate factors among women of reproductive age in Lao PDR: evidence from a nationally representative survey. Anemia. (2021) 2021:1–9. 10.1155/2021/8823030 PMC7822650 33520310

[31] Khan JR Awan N Misu F. Determinants of anemia among 6–59 months aged children in Bangladesh: evidence from nationally representative data. BMC Pediatr. (2016) 16(1):1–12. 10.1186/s12887-015-0539-9 26754288 PMC4707771

[32] Pasricha S-R Black J Muthayya S Shet A Bhat V Nagaraj S Determinants of anemia among young children in rural India. Pediatrics. (2010) 126(1):e140–e9. 10.1542/peds.2009-3108 20547647

[33] Smith LC Ruel MT Ndiaye A. Why is child malnutrition lower in urban than in rural areas? Evidence from 36 developing countries. World Dev. (2005) 33(8):1285–305. 10.1016/j.worlddev.2005.03.002

[34] Choi H-J Lee H-J Jang HB Park JY Kang J-H Park K-H Effects of maternal education on diet, anemia, and iron deficiency in Korean school-aged children. BMC Public Health. (2011) 11(1):1–8. 10.1186/1471-2458-11-1 22087564 PMC3250969

[35] Bazzocchi C Mortarino M Grandi G Kramer L Genchi C Bandi C Combined ivermectin and doxycycline treatment has microfilaricidal and adulticidal activity against dirofilaria immitis in experimentally infected dogs. Int J Parasitol. (2008) 38(12):1401–10. 10.1016/j.ijpara.2008.03.002 18433753

[36] VanBuskirk KM Ofosu A Kennedy A Denno DM. Pediatric anemia in rural Ghana: a cross-sectional study of prevalence and risk factors. J Trop Pediatr. (2014) 60(4):308–17. 10.1093/tropej/fmu020 24728349

[37] Foote EM Sullivan KM Ruth LJ Oremo J Sadumah I Williams TN Determinants of anemia among preschool children in rural, western Kenya. Am J Trop Med Hyg. (2013) 88(4):757. 10.4269/ajtmh.12-0560 23382166 PMC3617865

[38] Simbauranga RH Kamugisha E Hokororo A Kidenya BR Makani J. Prevalence and factors associated with severe anaemia amongst under-five children hospitalized at bugando medical centre, Mwanza, Tanzania. BMC Hematol. (2015) 15(1):1–9. 10.1186/s12878-015-0033-5 26464799 PMC4603816

[39] Gebreegziabiher G Etana B Niggusie D. Determinants of anemia among children aged 6–59 months living in kilte awulaelo woreda, Northern Ethiopia. Anemia. (2014) 2014:1–9. 10.1155/2014/245870 PMC4180192 25302116

[40] Nambiema A Robert A Yaya I. Prevalence and risk factors of anemia in children aged from 6 to 59 months in Togo: analysis from Togo demographic and health survey data, 2013–2014. BMC Public Health. (2019) 19(1):1–9. 10.1186/s12889-019-6547-1 30786883 PMC6383221

[41] Amegbor PM. Early-life environmental exposures and anaemia among children under age five in Sub-Saharan Africa: an insight from the demographic & health surveys. Sci Total Environ. (2022) 832:154957. 10.1016/j.scitotenv.2022.154957 35367541

[42] De Oliveira J Ventura S Souza AM Marchini JS. Iron deficiency anemia in children: prevalence and prevention studies in Ribeirão Preto, Brazil. Arch Latinoam Nutr. (1997) 47(2 Suppl 1):41–3. 9659418

[43] Eussen S Alles M Uijterschout L Brus F Van Der Horst-graat J. Iron intake and status of children aged 6-36 months in Europe: a systematic review. Ann Nutr Metab. (2015) 66(2–3):80–92. 10.1159/000371357 25612840

[44] Quizhpe E San Sebastián M Hurtig AK Llamas A. Prevalence of anaemia in schoolchildren in the Amazon area of Ecuador. Rev Panam Salud Publica. (2003) 13(6):355–61. 12880515 10.1590/s1020-49892003000500003

[45] Bhutta ZA Berkley JA Bandsma RH Kerac M Trehan I Briend A. Severe childhood malnutrition. Nat Rev Dise Primers. (2017) 3(1):1–18. 10.1038/nrdp.2017.67 PMC7004825 28933421

[46] Fabunmi T Onabanjo O Oguntona E Keshinro O Onabanjo J Obanla O Nutrient intakes and nutritional status of mothers and their under-five children in a rural community of Oyo state, Nigeria. Int J Child Health Nutr. (2013) 2(1):39–49.

[47] Greenwood B. The epidemiology of malaria. Ann Trop Med Parasitol. (1997) 91(7):763–9. 10.1080/00034983.1997.11813201 9625932

[48] Smith H. Prospects for the control of neglected tropical disease by mass drug administrative. Expert Rev Anti-Infect Ther. (2009) 7(1):37–56. 10.1586/14787210.7.1.37 19622056

[49] Collins-Andrews B McQuilkin P Udhayashankar K Adu E Moormann A. Presentation and treatment outcomes of Liberian children age 5 years and under diagnosed with severe malaria. Global Pediatric Health. (2019) 6:2333794X19884818. 10.1177/2333794X19884818 31700947 PMC6826913

[50] Muchie KF. Determinants of severity levels of anemia among children aged 6–59 months in Ethiopia: further analysis of the 2011 Ethiopian demographic and health survey. BMC Nutr. (2016) 2(1):1–8. 10.1186/s40795-016-0093-3

[51] Gebremeskel MG Mulugeta A Bekele A Lemma L Gebremichael M Gebremedhin H Individual and community level factors associated with anemia among children 6–59 months of age in Ethiopia: a further analysis of 2016 Ethiopia demographic and health survey. PLoS One. (2020) 15(11):e0241720. 10.1371/journal.pone.0241720 33186370 PMC7665792

[52] Tengco LW Rayco-Solon P Solon JA Sarol JN Jr Solon FS. Determinants of anemia among preschool children in the Philippines. J Am Coll Nutr. (2008) 27(2):229–43. 10.1080/07315724.2008.10719695 18689554

[53] Rao S Swathi P Unnikrishnan B Hegde A. Study of complementary feeding practices among mothers of children aged six months to two years-A study from coastal south India. Australas Med J. (2011) 4(5):252. 10.4066/AMJ.2011.607 23393516 PMC3562932

[54] Anticona C San Sebastian M. Anemia and malnutrition in indigenous children and adolescents of the Peruvian Amazon in a context of lead exposure: a cross-sectional study. Glob Health Action. (2014) 7(1):22888. 10.3402/gha.v7.22888 24560254 PMC3925814

[55] Ahmed T Hossain M Sanin KI. Global burden of maternal and child undernutrition and micronutrient deficiencies. Ann Nutr Metab. (2012) 61(Suppl 1):8–17. 10.1159/000345165 23343943

[56] Kothari MT Coile A Huestis A Pullum T Garrett D Engmann C. Exploring associations between water, sanitation, and anemia through 47 nationally representative demographic and health surveys. Ann N Y Acad Sci. (2019) 1450(1):249–67. 10.1111/nyas.14109 31232465 PMC6771505

[57] Habyarimana F Zewotir T Ramroop S. Spatial distribution and analysis of risk factors associated with Anemia among women of reproductive age: case of 2014 Rwanda demographic and health survey data. Open Public Health J. (2018) 11(1). 10.2174/1874944501811010425

[58] Nankinga O Aguta D Kabahuma C. Trends and determinants of anemia in Uganda: further analysis of the demographic and health surveys. DHS Working Papers. (2019) (149). 10.1186/s12889-019-8114-1 PMC6937990 31888579

[59] Gujo AB Kare AP. Prevalence of intestinal parasite infection and its association with Anemia among children aged 6 to 59 months in sidama national regional state, Southern Ethiopia. Clin Med Insights Pediatr. (2021) 15:11795565211029259. 10.21203/rs.3.rs-2265012/v1 34276235 PMC8255584

[60] Gebremeskel MG Tirore LL. Factors associated with Anemia among children 6–23 months of age in Ethiopia: a multilevel analysis of data from the 2016 Ethiopia demographic and health survey. Pediatric Health Med Ther. (2020) 11:347. 32982542 10.2147/PHMT.S258114 PMC7508559

[61] Ayele D Zewotir T Mwambi H. Modelling the joint determinants of a positive malaria rapid diagnosis test result, use of mosquito nets and indoor residual spraying with insecticide. Occup Health South Afr. (2014) 20(4):20–7.

[62] Gaston RT Ramroop S Habyarimana F. Joint modelling of malaria and anaemia in children less than five years of age in Malawi. Heliyon. (2021) 7(5):e06899. 10.1016/j.heliyon.2021.e06899 34027150 PMC8121655

[63] Zgambo M Mbakaya BC Kalembo FW. Prevalence and factors associated with malaria parasitaemia in children under the age of five years in Malawi: a comparison study of the 2012 and 2014 malaria indicator surveys (MISs). PLoS One. (2017) 12(4):e0175537. 10.1371/journal.pone.0175537 28399179 PMC5388476

[64] Fosu MO Frimpong F Arthur M. Factors associated with haemoglobin prevalence among Ghanaian children aged 6–59 months. J Biology Agric Healthc. (2014) 4(2):132–40.

[65] Hakizimana D Nisingizwe MP Logan J Wong R. Identifying risk factors of anemia among women of reproductive age in Rwanda–a cross-sectional study using secondary data from the Rwanda demographic and health survey 2014/2015. BMC Public Health. (2019) 19(1):1–11. 10.1186/s12889-019-8019-z 31829161 PMC6907339

[66] Gebremedhin S Asefa A. Association between type of contraceptive use and haemoglobin status among women of reproductive age in 24 Sub-Saharan Africa countries. BMJ Sex Reprod Health. (2019) 45(1):54–60. 10.1136/bmjsrh-2018-200178 30463847

[67] Correa-Agudelo E Kim H-Y Musuka GN Mukandavire Z Miller FD Tanser F The epidemiological landscape of anemia in women of reproductive age in Sub-Saharan Africa. Sci Rep. (2021) 11(1):1–10. 10.1038/s41598-021-91198-z 34099773 PMC8184956

